# A Review of PRDM13 Transcriptional Regulation in Vertebrate Retinal Development and Disease

**DOI:** 10.1167/iovs.67.12.33

**Published:** 2026-10-16

**Authors:** Emily R. Nettesheim, Katherine J. Wert

**Affiliations:** 1 Department of Ophthalmology, University of Texas Southwestern Medical Center, Dallas, Texas, United States; 2 Department of Molecular Biology, University of Texas Southwestern Medical Center, Dallas, Texas, United States; 3 Peter O'Donnell Jr. Brain Institute, University of Texas Southwestern Medical Center, Dallas, Texas, United States; 4 Hamon Center for Regenerative Science and Medicine, University of Texas Southwestern Medical Center, Dallas, Texas, United States

**Keywords:** PRDM13, North Carolina macular dystrophy (NCMD), neuronal development, neural development, maculopathies

## Abstract

The retina is a complex neuronal tissue whose development from a common retinal progenitor cell population is tightly controlled by a complex gene regulatory network driven by transcription factors. PRDM13 is a transcription factor known to play a critical role in the specification of neurons across the central nervous system, and its expression is maintained into adulthood in a limited number of neuronal types, including neurons of the dorsomedial hypothalamus and retinal amacrine cells. Dysregulation of *PRDM13* has been associated with three inherited human maculopathies – North Carolina macular dystrophy (NCMD), progressive bifocal chorioretinal atrophy (PBCRA), and congenital posterior polar chorioretinal hypoplasia (CPPCRH) – indicating that PRDM13 may also play a role in development and maintenance of non-amacrine retinal cell types. Herein, we review the known roles of PRDM13 across the developing central nervous system to provide a comprehensive understanding of PRDM13’s general and tissue-specific functions, as well as how PRDM13 dysregulation may lead to improper macular development and disease. Future studies into PRDM13 and its downstream regulatory network will be important to provide critical insights into the regulation of cell fate decisions in the developing retina.

The murine retina is a complex neuronal tissue composed of over 100 subtypes of neurons, which must be specified and synapsed correctly to allow propagation of visual signal to the brain.^1^^–^^4^ The cells of the retina arise from a common retinal progenitor cell population under control of a complex gene regulatory network driven by transcription factors.^4^^–^^7^ Thus, the understanding of transcriptional regulation in retinal formation and health is of high importance. PRDM13 is a transcription factor known to play a critical role in the specification and patterning of neurons within the developing dorsal neural tube, brain, and amacrine cells of the retina. In mature tissues, PRDM13 is selectively expressed within neurons of the dorsomedial hypothalamus and in some subtypes of amacrine cells within the retina.^8^^–^^13^ PRDM13 dysregulation has been associated with three human macular degenerative diseases – North Carolina macular dystrophy (NCMD), progressive bifocal chorioretinal atrophy (PBCRA), and congenital posterior polar chorioretinal hypoplasia (CPPCRH) – which primarily effect the retinal pigment epithelium (RPE) and photoreceptors.^14^^–^^16^ This suggests that there may be an unknown role for PRDM13 transcriptional regulation in the retina for development and maintenance of non-amacrine cell populations. In this article, we review PRDM13 activity across the developing vertebrate central nervous system, with a focus on using this extended range of knowledge to better understand the retina-specific functions of PRDM13; providing critical insights into the regulation of cell fate decisions in the developing retina and how PRDM13 dysregulation could lead to a spectrum of inherited retinal dystrophies.

## PR Domain Containing Protein Transcriptional Regulators

The PR Domain Containing Protein (PRDM) family of transcription factors has been associated with genetic regulation of critical biological processes from development to cancer (Fig. 1).^17^^,^^18^ The members of this family are characterized by the presence of a Su(var)3–9, Enhancer-of-zeste and Trithorax (SET)-like domain termed the PR/SET domain. The PR/SET domain is a hybrid of two previously identified domains, the positive regulatory domain I-binding factor 1 (PRDF1) and retinoblastoma protein-interacting zinc finger protein 1 (RIZ1), and conveys histone methyltransferase (HMT) activity to 5 members of the PRDM family – PRDM2, 3, 8, 9, and 16.^19^ Other members of the PRDM family utilize this PR/SET domain to recruit histone modifiers.^18^ Why some members of the PRDM family have intrinsic HMT activity while others do not has yet to be fully understood; however, review of the PR/SET domain sequence across PRDM family members shows that not all residues are conserved.^17^^,^^18^ Investigation into three distinct residues in the PR/SET domain which differ between PRDM7 and PRDM9 – two PRDM family members which have varying HMT catalytic activity – revealed that these residues affect catalytic function and substrate selectivity.^20^ Therefore, it is likely that as the many PRDM family members evolved their tissue specific roles, they acquired mutations which are beneficial for their function, but may be at the expense of retaining intrinsic HMT activity. Further characterization of PRDM crystal structures and interactors will be necessary to fully understand how mutated residues within the PR/SET domain alter HMT activity for PRDM family members. Comparison of PR/SET domain sequences, available crystal structures, and known binding partners have been extensively reviewed.^17^^–^^19^^,^^21^

**Figure 1.**
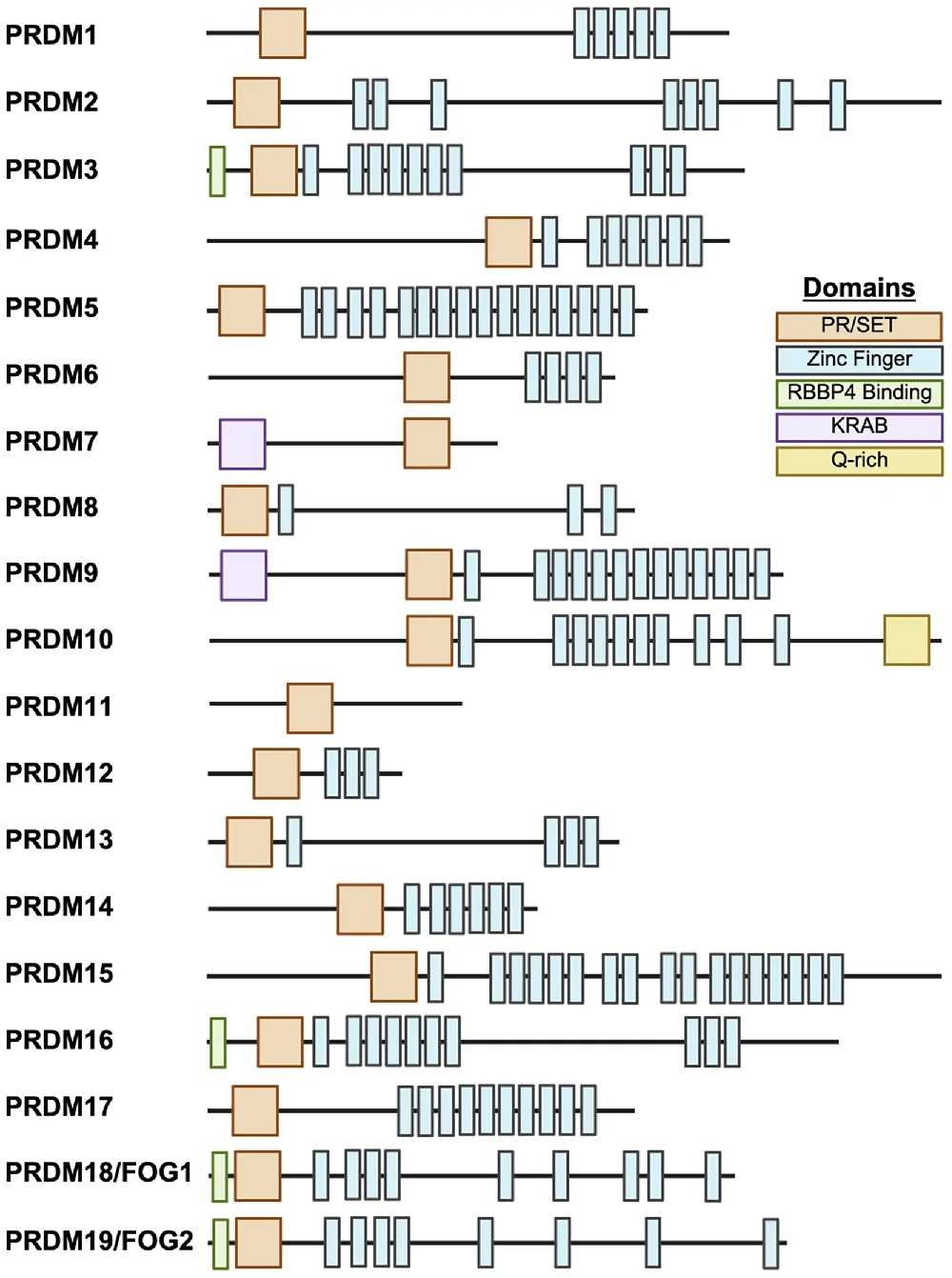
**PRDM family of transcription factors.** There are 19 transcription factors in the PRDM family. Although all family members contain one PR/SET domain, each family member contains a variable number of zinc finger, RBBP4, KRAB, and Q-rich domains.^17^ Figure generated in BioRender (agreement number: EX2A1D5TOO).

In addition to the PR/SET domain, each member of the PRDM family, with the exception of PRDM11 and PRDM7, has a variable number of Cys2-His2 zinc finger domains which can be used for DNA, RNA and protein binding, allowing PRDM family members to perform a diverse set of functions.^18^ Twelve of the 19 PRDM family members have established functions within the central nervous system.^19^ Within the vertebrate retina, PRDM1, PRDM8, PRDM13, and PRDM16 have been shown to play critical roles in the development and maintenance of photoreceptor, bipolar, and ganglion cells.^9^^,^^11^^,^^22^^–^^30^

PRDM13 is composed of the PR/SET domain and four zinc finger domains (Fig. 2A).^19^ During development, the primary function of PRDM13 is the specification and maintenance of neurons. There is conflicting evidence as to whether PRDM13 has inherent methyltransferase activity, as PRDM13 was shown to cause H3K9 and H3K27 trimethylation in an HEK293 in vitro assay; however, assays with truncated PRDM13 proteins suggest that only the zinc finger domains are necessary to execute its currently understood functions in development of the neural tube, retina, and neuroectoderm.^8^^,^^11^^,^^31^^,^^32^ Overall, literature has shown that the loss of PRDM13 is linked to cerebellar hypoplasia and perinatal brainstem dysfunction, whereas the overexpression of PRDM13 is linked to multiple retinal diseases.^12^^,^^14^^–^^16^^,^^33^^–^^37^ Computational analysis to determine potential genes co-regulated with PRDM13 imply a role in regulation of cell cycle, neuronal differentiation, and GABAergic neuron specification and migration.^38^

**Figure 2.**
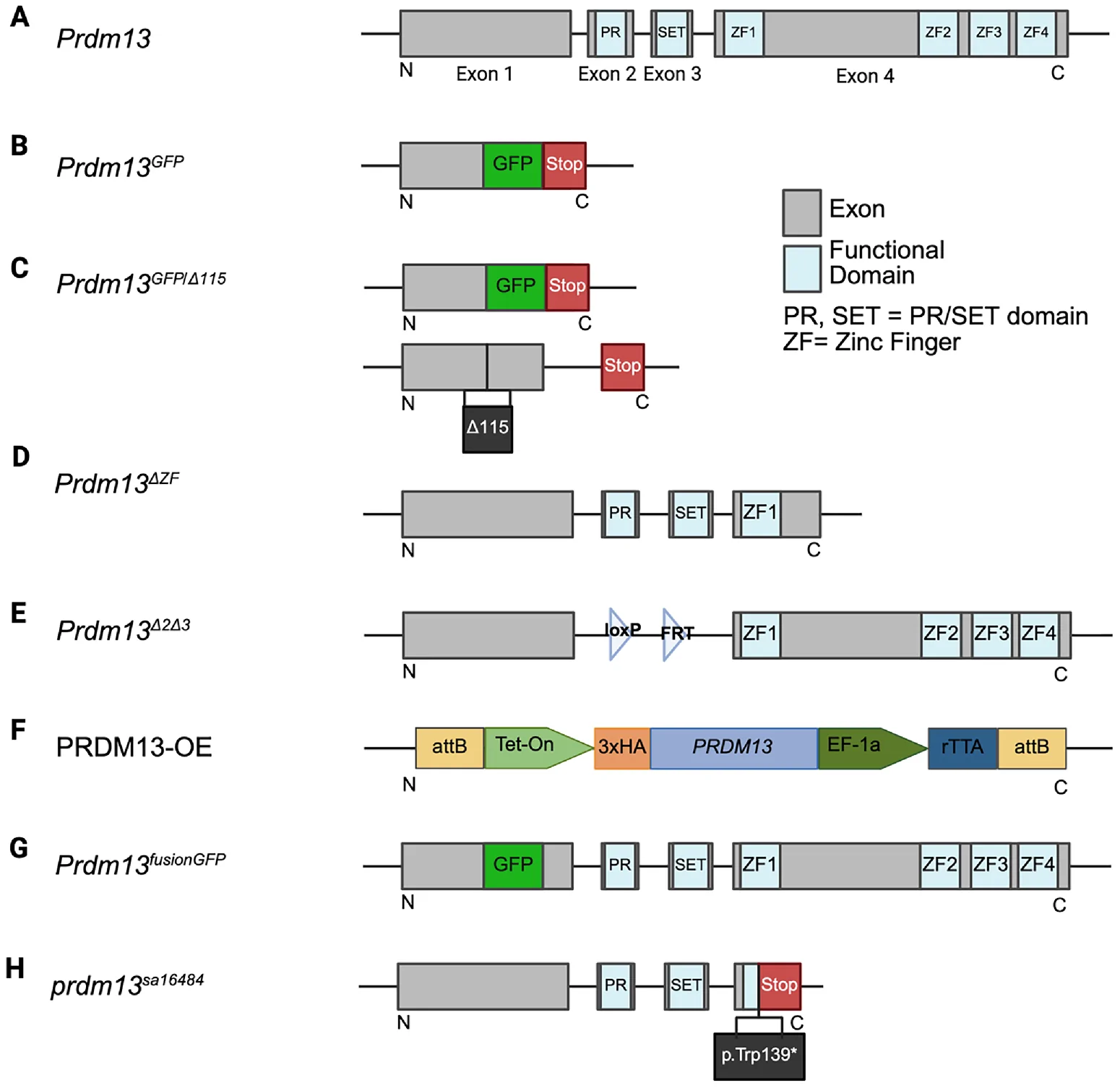
**PRDM13 exon structure and transgenic models.** (**A**) PRDM13 is composed of four exons.^16^ The PR/SET domain spans exons two and three, while the four zinc finger (ZF) domains are in exon four. (**B**) The *Prdm13^GFP^* mouse strain contains a green fluorescent protein (GFP) coding sequence followed by a stop codon knocked-in to the first exon of *Prdm13*.^10^ (**C**) The *Prdm13^GFP/^^Δ^^115^* mouse strain carries one allele matching the *Prdm13^GFP^* mouse strain in **B**, whereas the second allele contains an early stop codon in the second exon of *Prdm13*, resulting in a 115 bp deletion in exon one.^10^ (**D**) The *Prdm13^Δ^*^ZF^ mice have a truncated *Prdm13* gene which removes the three most C-terminal ZF domains.^10^ (**E**) The *Prdm13^Δ^^2^^Δ^^3^* mice used the cre-*loxP* system to remove exons 2 and 3 of the *Prdm13* coding sequence.^11^ (**F**) The PRDM13-OE mouse strain does not impact the endogenous *Prdm13* locus, but rather has a tetracycline-inducible *PRDM13* gene knocked into the *Rosa26* safe harbor locus.^21^ (**G**) The *Prdm13^fusionGFP^* mouse strain has the GFP coding sequence knocked-in to the first exon of *Prdm13*. This knock-in is in-frame and does not result in the loss of PRDM13 but rather acts as a tag on the PRDM13 protein.^10^ (**H**) A transgenic zebrafish strain, *prdm13^sa16484^*, has point mutations located within the first ZF domain which generates a truncated PRDM13 protein that does not contain any of the ZF domains. Figure generated in BioRender (agreement number: FN2A1D5ZGL).

## Vertebrate Models to Study PRDM13

To dive deeper into PRDM13’s regulation of neuronal health and development, multiple mouse models have been generated. The first mouse model has green fluorescent protein (GFP) followed by a stop codon knocked-in to the first exon of *Prdm13* (*Prdm13^GFP^*; Fig. 2B).^10^ This *Prdm13^GFP^* mouse was found to be neonatal lethal, so a heterozygous model was developed. In this model, one allele contains the same GFP and stop codon knocked-in to the first exon of *Prdm13* and the other allele contains a 115 base pair (bp) deletion within the first exon of *Prdm13*, creating a frameshift in the second exon of *Prdm13* that was expected to cause an early stop codon and loss of PRDM13 protein (*Prdm13^GFP/^^Δ^^115^*; Fig. 2C).^9^^,^^10^ However, the *Prdm13^GFP/^^Δ^^115^* mouse model is able to avoid the neonatal lethality as the 115 bp deletion appears to create a hypomorphic allele in which a mutated PRDM13 protein is expressed that includes the C-terminal zinc finger. The production of this mutated protein is sufficient to allow for prolonged study of the impact of PRDM13 loss on neuronal development and maturation.^10^

In addition to these two PRDM13 knockout mouse models, strains have also been developed with deletions in the *Prdm13* gene. The *Prdm13^Δ^^ZF^* mouse contains a truncated *Prdm13* gene which no longer retains the three most C-terminal zinc finger domains. This model was reported to phenocopy the *Prdm13^GFP^* mouse model (Fig. 2D).^10^ Another *Prdm13* truncation mouse model was developed utilizing the Cre-*loxP* system to remove exons 2 and 3 of *Prdm13* (*Prdm13^Δ^^2^^Δ^^3^*; Fig. 2E) which encode for the PR/SET domain.^11^ Although the *Prdm13^Δ^^2^^Δ^^3^* mouse has been characterized as a knockout model, the zinc fingers – the known functional domains of PRDM13 – were not disrupted. Therefore, it is particularly important to consider this model within the context of other PRDM13 knockout mouse models to fully elucidate how PRDM13 loss impacts the retina and overall neuronal development.^11^

In addition to the many PRDM13 knockout/loss-of-function mouse lines, one mouse model has been developed which allows for inducible *PRDM13* overexpression.^26^ This model, termed PRDM13-OE, utilizes the TRE3G tetracycline-inducible promoter to drive expression of the human *PRDM13* gene with an N-terminal 3xHA tag. The reverse tetracycline-controlled transactivator (*rtTA*) expression is under control of the ubiquitous EF-1α promoter. Both the *3xHA-PRDM13* gene and *rtTA* were knocked-in to the *Rosa26* safe harbor locus, with no disruption to the endogenous mouse *Prdm13* gene. In this model, when a tetracycline is provided to the PRDM13-OE mice, human *PRDM13* is expressed ubiquitously alongside endogenous *Prdm13* expression allowing for the study of *PRDM13* overexpression in a controlled manner (Fig. 2F).^26^

As there are no reliable commercial antibodies for PRDM13, a GFP tagged mouse has also been developed, with the GFP coding sequence knocked-in to the first exon of *Prdm13*. There is no stop codon in this mouse model, allowing for production of the full PRDM13 protein with a GFP tag (*Prdm13^fusionGFP^*; Fig. 2G).^10^ A zebrafish mutant, *prdm13*^sa16464^, is also available through the Zebrafish Mutation Project. This fish contains a single point mutation in Prdm13 at amino acid 139 which creates a premature stop codon part way through the first zinc finger domain, causing for a truncated protein (Fig. 2H).^33^^,^^39^ In homozygous mutant fish, abnormal body curvature and lower jaw morphology were noted, and there is lethality between 7 and 9 days post fertilization (dpf). This mutation mimics the mutations identified in patients with perinatal brainstem dysfunction described by Coolen et al.^33^ With these various transgenic models, as well as additional in vitro model systems, research advances have been made into the role of PRDM13 in neuronal development, as discussed in the following section (Table 1).

**Table 1.** Cellular Changes in PRDM13 Transgenic Model Systems

Model System	Species	Genetic Background	Study Tissue	Key Findings
*Prdm13^GFP^*	Mouse	*Prdm13* null due to GFP insertion in exon 1 followed by a stop codon. Leads to neonatal lethality.	Neural tube	- Loss of *Pax2+* neurons, increase in *Tlx1/3+* neurons - Misexpression of ventral neural tube factors, *Olig2* and *Prdm12*, in the dorsal neural tube
			Cerebellum	- E16.5 – Reduction in *Pax2+* inhibitory neurons, increase in *Tlx3+* excitatory neurons - E17.5 – reduced overall size and increased number of apoptotic cells - Reduction and improper localization of Purkinje cells
*Prdm13^GFP/^^Δ^^115^*	Mouse	One allele from *Prdm13^GFP^* model, second allele is a *Prdm13* hypomorphic allele created by a 115 bp deletion in the first exon of *Prdm13* which was originally predicted to create an early stop codon.	Retina	- Reduced population of amacrine cells in the mature retina - E17.5-P2 – no change in *Pax6* expression - P5 – significant reduction in *Ebf3+* amacrine cells, increase in *Prdm13+* cells expressing *Bhlhb5* - Loss of S3 synapse layer in IPL
*Prdm13^Δ^^ZF^*	Mouse	Truncation of *Prdm13* resulting in loss of zinc fingers 2, 3, and 4.	Neural tube	- Reported to phenocopy *Prdm13^GFP^* mice
*Prdm13^Δ^^2^^Δ^^3^*	Mouse	cre-*loxP* system used to delete exons 2 and 3 of *Prdm13*.	Cerebellum	- P5 – Signs of cerebellar hypoplasia - Reduction in *Pax2+* cells, increase in *Tlx3+* cell population - No change to Purkinje cell population
			Hypothalamus	- By E14.5, reduction of *Kiss1+* neurons in Arc nucleus
			Retina	- Reduction of amacrine cell population in mature retina - Loss of S3 synapse layer in IPL - Elevated sensitivity of optokinetic response
PRDM13-OE	Mouse	Doxycycline inducible human *PRDM13* gene knocked in to the *Rosa26* safe harbor locus; *Prdm13* locus is not mutated.	Retina	- Acute *PRDM13* overexpression leads to photoreceptor cell death but no change in retinal function - Greater than 6 days of *PRDM13* overexpression led to significant photoreceptor-specific cell loss and reduced visual response on ERG - No detectable change in the amacrine cell population
*Prdm13^fusionGFP^*	Mouse	GFP inserted into exon 1 of *Prdm13*.	Neural tube	- Utilized for ChIP-seq assay, identified common motifs at PRDM13 binding sites and associated potential downstream regulatory targets
			Retina	- E13.5 – all PRDM13+ cells colocalize with PTF1a - E15.5 – 89% of PRDM13+ cells express PTF1a; PRDM13 co-localizes with a small number of EdU+ cells - P0 – 80% PRDM13+ cells express PTF1a - Adulthood – PRDM13 expressed in about 33% of amacrine cells, primarily nGnG subtypes - Adulthood – PRDM13 excluded from cholinergic, dopaminergic and glutamatergic subtypes
*prdm13^sa16464^*	Zebrafish	Point mutation, p.Trp139, creates a stop codon in the first zinc finger domain, leading to truncation of prdm13 and loss of all zinc fingers. Lethality observed by 7 dpf.	Cerebellum	- *calb+* eurydendroid cells unaffected - *pvalb7+* Purkinje cells reduced
			Posterior Hindbrain	- *lhx1+* ION neurons population is lost

Transgenic vertebrate models which knockout, truncate, or overexpress PRDM13 have provided critical insights into the role of PRDM13 throughout the central nervous system.^9^^–^^11^^,^^26^^,^^33^

## PRDM13 in Vertebrate Neuronal Development

### PRDM13 Expression in Early Developing Neurons

The timing of PRDM13 expression within the central nervous system has been studied in multiple vertebrate models.^11^^,^^16^^,^^31^^,^^33^^,^^40^ The earliest expression of *PRDM13* in human samples was detected by real-time polymerase chain reaction (RT-PCR) analysis of human induced pluripotent stem cells (iPSCs) undergoing differentiation into retinal eyecup-like structures, where *PRDM13* expression was shown at day 0 of differentiation.^16^ The level of *PRDM13* expression slowly decreased over the first 100 days of differentiation, with some *PRDM13* expression remaining at this terminal timepoint.^16^ *PRDM13* can be detected by in situ hybridization in the dorsal neural tube and dorsal ventral hindbrain regions as early as Carnegie stage 13, the equivalent of approximately 32 days post conception (dpc) in human development.^33^ Sequencing performed on human fetal retina samples at 59, 82, and 125 dpc found that PRDM13 marks a population of cells transitioning from the general neurogenic precursor population toward the mature amacrine and horizontal cell fate.^40^ This population was identified at all three timepoints examined and found to be in generally equal proportion across time.^40^

Within developing zebrafish, *prdm13* expression is detectable via in situ hybridization and single cell RNA sequencing as early as 24 hours post fertilization (hpf) in rhombomeres 2 to 7, with increased intensity in the ventricular zone and HuC expressing neurons within rhombomere 7, as well as the pharyngeal arch cells.^33^ By 48 hpf, *prdm13* can be detected in the cerebellar primordium.^33^ In the developing frog, zygotic *Prdm13* expression is detected as early as stages 12 and 13, the approximate time in which the neural plate becomes identifiable and the eye field is first specified.^31^^,^^41^^,^^42^ In situ hybridization for *Prdm13* in developing frogs shows distinct expression throughout the neural fold stages, with localization to the dorsal portion of the posterior hindbrain and spinal cord upon neural tube closure. Expression within the retina was first noted at stage 28, just before patterning of the central nervous system begins.^31^^,^^41^ Within the mouse, immunohistochemistry (IHC) performed at embryonic day (E)9.5, E10.5, and E13.5 shed light on PRDM13 expression throughout the early developing central nervous system.^43^ At E9.5, PRDM13 expression was noted in the craniofacial structures, developing somites, brain – specifically the telencephalon, tegmentum, cerebellum, midbrain, dorsal root ganglia, and hindbrain – and notochord. Expression at E10.5 was similar, with the authors’ noting additional expression in the tegmentum and especially high levels of expression in the spinal cord.^43^ PRDM13 expression was not detected within the optic vesicle until E13.5.^43^ The results of this IHC experiment are consistent with a separate study that published *Prdm13* in situ hybridization performed on developing mouse eye tissue, in which *Prdm13* was not expressed in developing eye tissue at E11.5 but was present by E12.5.^11^

### PRDM13 in Neuroectoderm Differentiation

Research into the role of PRDM13 in cell fate decisions at the earliest stage of neural development, the specification of the neuroectoderm from mouse pluripotent stem cells, has revealed key insights into the utility of the various zinc finger domains of PRDM13.^32^ Basinski et al. developed a reporter system which knocked-in GFP to the endogenous *Rax/RX* allele and mCherry into the *Sox1* allele of mouse embryonic stem cells (mESCs).^32^ This allowed them to track expression of these two markers, where *Rax/RX* is specifically expressed in neuroectoderm specifying to an eye field fate, whereas *Sox1* is specifically expressed in non-eye field neuroectoderm. The authors then differentiated their reporter lines using two protocols, one which will form eye-field mouse neuroectoderm organoids (mNEOs) and one which forms non-eye-field mNEOs. *Prdm13* expression was induced on day 1 of the differentiation protocols when the mESCs are first exiting pluripotency, and analysis was performed 5 days later. PRDM13 was found to repress the specification of mESCs to eye-field mNEOs, whereas the *Sox1* expression was increased. This work demonstrated that PRDM13 plays a contrasting role in the specification of eye-field and non-eye field neuroectoderm.^32^

Utilizing bulk RNA sequencing, Basinski et al. identified upregulation of key players in the WNT/β-catenin pathway following induced *Prdm13* expression in the eye-field mNEOs.^32^ Inhibition of the WNT signaling pathway via small molecule inhibitors relieved the repressive effect of induced *Prdm13* expression on the differentiation of eye-field mNEOs. This suggests that during the earliest stages of neuronal development, PRDM13 regulates members of the WNT signaling pathway.^32^

Additionally, the authors set out to determine which of the PRDM13 functional domains, the PR domain or its four zinc finger domains, may be responsible for the regulatory function of PRDM13. The authors generated plasmids containing various truncated forms of *Prdm13* and constructs where each zinc finger was inactivated individually. These constructs were used in conjunction with the reporter line previously described and each construct was evaluated for their effect on the differentiation of eye-field mNEOs. This assay determined that zinc fingers 1 and 2 are required for the repression of eye-field mNEO differentiation from mESCs.^32^

### PRDM13 in Neural Tube Development

Alongside a new role for PRDM13 in the onset of neuroectoderm development, studies in multiple vertebrate systems have examined the role of PRDM13 in neural tube development (Fig. 3).^8^^,^^10^^,^^31^ *Prdm13* expression within the developing neural tube is generally conserved across species and was found to be associated with *Ptf1a* expression in chick, frog, and mouse.^8^^,^^10^^,^^31^ ChIP-seq for PTF1a on mouse dorsal neural tube tissue identified several PTF1a binding sites near to *Prdm13*, suggesting that *Prdm13* is a downstream regulatory target of PTF1a.^8^ Critical work to define the role of Prdm13 in neuronal development was published in a chick study, where Prdm13 was found to be an important regulator in fate specification between excitatory and inhibitory neurons.^8^ Studies involving overexpression and knockdown of both *Ptf1a* and *Prdm13* within the chick neural tube found that Prdm13 is both necessary and sufficient to promote the inhibitory GABAergic cell fate over the excitatory glutamatergic cell fate.^8^^,^^10^

**Figure 3.**
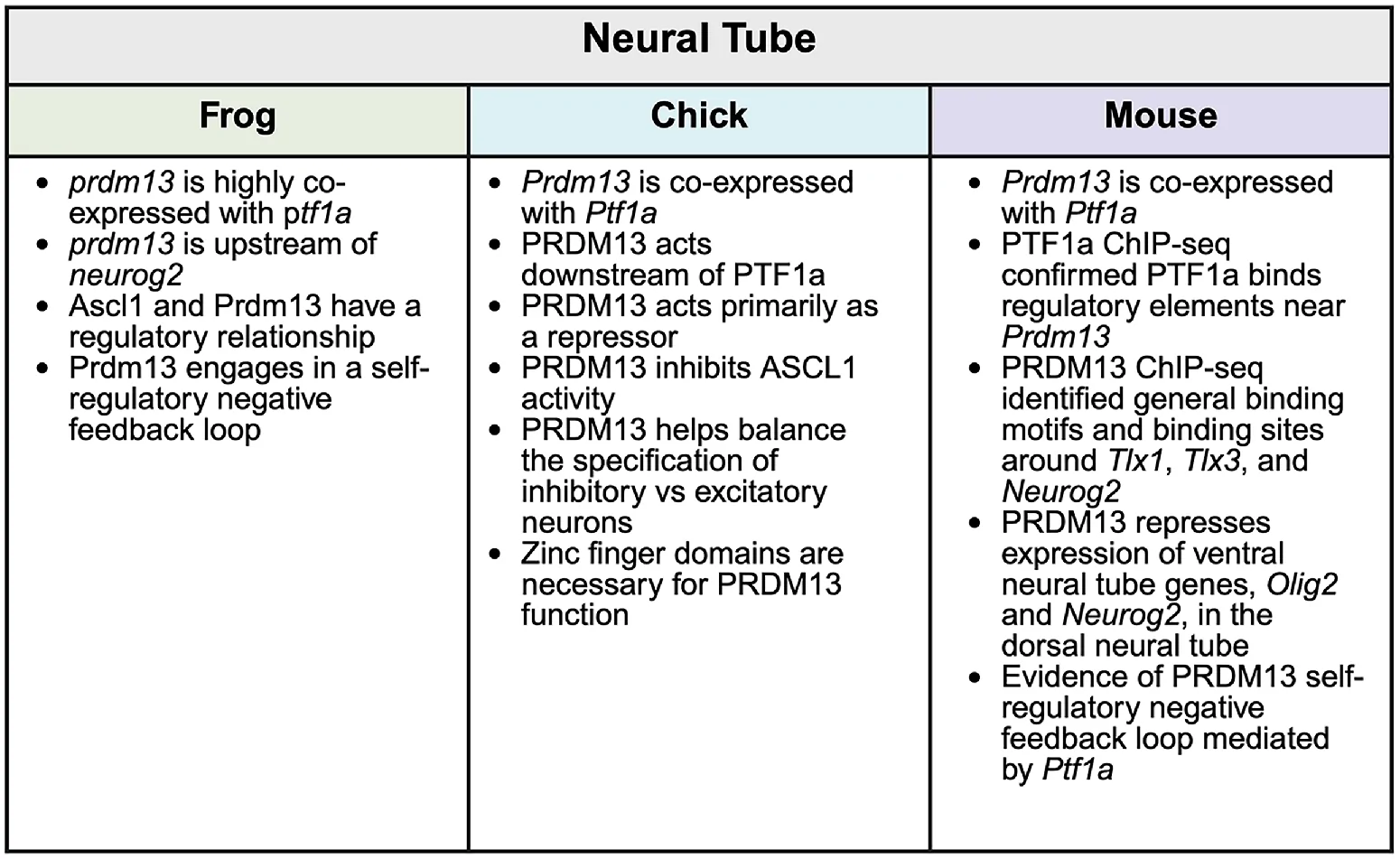
**Summary of PRDM13 function in the neural tube across vertebrates.** PRDM13 has been extensively studied in the neural tube across multiple vertebrate species: frog, chick, and mouse.^8^^,^^10^^,^^31^ These studies show strong conservation of PRDM13 expression and function across species. Particularly, PRDM13 is often co-expressed with PTF1a, engages in a self-regulatory feedback loop, and regulates gene expression critical for proper specification and patterning of neurons. Figure generated in BioRender (agreement number: RF2A1D62ZQ).

Studies conducted in the chick neural tube to express truncated forms of Prdm13, one without a PR domain and one without the zinc fingers, demonstrated that the zinc finger domains were necessary for proper Prdm13 function, whereas the PR domain was not.^8^ Constructs containing Prdm13 fused to either the VP16 activator domain or the engrailed repressor domain were ectopically expressed in the chick neural tube and the resulting populations of Tlx1/3 expressing and Pax2 expressing neurons were assessed.^8^ As the Prdm13 tagged with the engrailed repressor domain was nearly identical to that of wildtype, the authors’ identified that Prdm13 acts primarily as a repressor of downstream target genes. Therefore, Prdm13 is thought to regulate the balance of excitatory and inhibitory neuronal cell fates through the repression of *Tlx1/3*, which derepresses *Pax2* and in turn promotes inhibitory cell specification while repressing excitatory cell specification (Fig. 4).^8^

**Figure 4.**
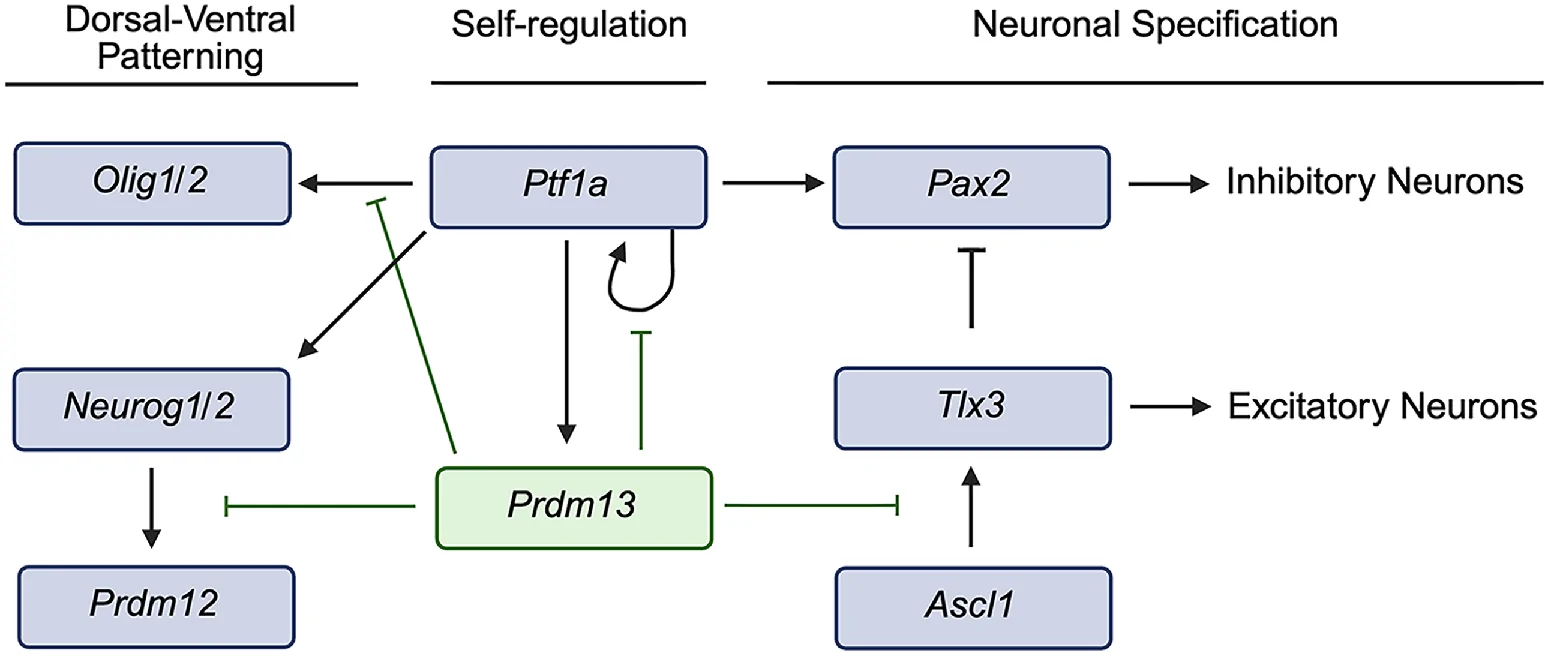
**Known PRDM13 regulatory network in the developing central nervous system.** PRDM13 has been shown to act as a repressor, with three primary roles in developing neurons of the central nervous system: ensuring proper expression of genes in the dorsal neural tube, a negative-feedback loop to self-regulate its own expression, and balancing expression of excitatory and inhibitory neuron specification.^8^^,^^10^^,^^31^^,^^44^ Figure generated in BioRender (agreement number: DI2A1D6A09).

Additionally, as Ascl1 was known to have opposing regulatory activity to Ptf1a – driving *Tlx1/3* expression to promote excitatory neuron formation – the authors overexpressed both *Ascl1* and *Prdm13* in the chick neural tube and assessed the impact on neuronal specification.^8^ They found that when co-expressed, there was a 3.3-fold reduction in the number of Tlx1/3 expressing neurons. Ectopic *Prdm13* expression was found to not affect *Ascl1* gene expression, suggesting that Prdm13 does not inhibit *Ascl1* expression, but does inhibit its activity (see Fig. 4).^8^ Co-immunoprecipitation (Co-IP) performed in HEK293T cells confirmed a direct interaction between PRDM13 and ASCL1. Therefore, at sites where both PRDM13 and ASCL1 bind, such as *Tlx3*, it is likely that PRDM13 and ASCL1 form a complex which then represses gene expression.^8^

Investigation into the role of Prdm13 in the frog neural tube revealed a high degree of conservation of Prdm13 function across species. In the frog neural tube, Prdm13 was co-expressed with and downstream of Ptf1a.^31^ The authors also determined that in addition to Ptf1a being an activator of *prdm13* expression, ectopic expression of *ascl1* was sufficient to increase *prdm13* expression. Although the authors do not claim that Ascl1 directly regulates *prdm13*, these data further support the relationship between Ascl1 and Prdm13 in proper neuronal cell development within vertebrates.^31^ In addition to its role in excitatory versus inhibitory neuronal specification, ectopic expression of mouse PRDM13 was found to repress frog Prdm13 expression, suggesting that Prdm13 participates in a negative self-regulatory feedback loop.^31^ A later study conducted in the mouse dorsal neural tube further supported a self-regulatory function for PRDM13.^10^ Through ChIP-seq data and experimental validation, it was determined that PRDM13 binds the enhancer for *Ptf1a*, the known activator of *Prdm13*, thereby repressing *Ptf1a* expression which in turn regulates its own expression.^10^

ChIP experiments on mouse neural tubes have provided additional information about potential regulatory targets for PRDM13.^8^^,^^10^ Although there has yet to be a defined binding motif established for PRDM13, motif analysis from the ChIP data was able to identify common motifs where PRDM13 bound in the genome. This included E-BOX, RFX, PDX1, and SOX motifs. The E-box motif was the most commonly identified, matching 40% of PRDM13 binding sites. The top genes associated with PRDM13 binding sites include members of the *Olig*, *Phox*, *Prdm*, *Neurog*, and *Pou* families.^10^ *Neurog2*, an activator of *Tlx3*, identified as a direct regulatory target of Prdm13 in the frog neural tube, was also a hit within the mouse neural tube ChIP-seq dataset, suggesting high conservation of PRDM13 function across species.^10^^,^^31^ This indicates that PRDM13 likely acts through multiple mechanisms, not just ASCL1, to ensure the inhibition of the excitatory neuronal cell fate. The mouse ChIP-seq data also identified *Olig2* and *Prdm12*, factors typically expressed in the ventral neural tube, as potential regulatory targets of PRDM13.^10^ Further validation of these hits in the *Prdm13^GFP^* mice showed that a loss of PRDM13 leads to expression of *Olig2* and *Prdm12* in the dorsal neural tube, identifying a new function of PRDM13 in proper patterning of neurons by ensuring that ventral specific genes are not expressed dorsally.^10^

Overall, within the developing neural tube, PRDM13 has been found to play three critical roles: (1) Inhibition of the excitatory neuron cell fate to promote inhibitory neuron specification; (2) repression of ventral neural tube genes in the dorsal neural tube to ensure proper dorsal-ventral patterning; and (3) self-regulation of its own expression. The work to define these functions has been conducted in frogs, chicks, and mice, defining PRDM13 as a critical regulator of proper development within the vertebrate neural tube.^8^^,^^10^^,^^31^

### PRDM13 in Brain Development

PRDM13 has also been studied in the context of tissue-specific development. Investigation into the role of PRDM13 in the development of the cerebellum has established PRDM13 as a regulator of *Pax2* lineage specification.^44^ *Prdm13* expression begins at approximately E11.5 in the developing mouse cerebellum, where it is primarily expressed in cells of the ventricular zone. At E12.5, *Prdm13* is detected in progenitor cells expressing *Ptf1a*, *Ascl1*, *Olig2*, and a few co-expressing *Pax2*; but is absent from more mature cell types, such as those expressing *Tlx3* and *Calb1*. Histological analysis of cerebellar sections from *Prdm13^GFP^* mouse pups at E16.5 revealed a reduction in the number of *Pax2* inhibitory neurons and an increase in the number of *Tlx3* excitatory neurons.^44^ This is consistent with previous literature in the dorsal neural tube which found that PRDM13 is a regulator of the inhibitory versus excitatory cell fate decision of developing neurons.^8^^,^^10^ In particular, the loss of PRDM13 was found to not only decrease the number of Purkinje cells, an inhibitory neuron type, but also lead to the improper organization of the Purkinje cells that did develop. In contrast, the excitatory *Tbr1* cell population did not show a change in the total number of cells but was found to be mislocalized. By E17.5, the cerebellum of *Prdm13^GFP^* mice is notably smaller with an increased number of apoptotic cells.^44^

The role of *prdm13* in the developing cerebellum and posterior hindbrain has also been studied using the *prdm13*^sa16464^ transgenic zebrafish model.^33^^,^^39^ In homozygous *prdm13*^sa16464^ fish, Calb expressing eurydendroid cells were found to be unaffected.^33^ There was a reduced number of Pvalb7 expressing Purkinje cells, consistent with work conducted in *Prdm13^GFP^* mice, further stressing the critical role of PRDM13 for proper Purkinje cell specification.^33^^,^^44^ The *prdm13*^sa16464^ fish were also found to have a loss of the Lhx1 expressing ION neurons. The authors note that this phenotype is highly similar to the clinical pathology of children and fetuses with bi-allelic PRDM13 mutations.^33^

In the post-natal mouse brain, *Prdm13* is expressed in the prospective white matter of the developing cerebellum.^12^ A study of the post-natal brain by Whittaker et al. utilized the *Prdm13^Δ^^2^^Δ^^3^* mouse model and showed signs of cerebellar hypoplasia by post-natal day (P)5.^12^ This study also noted a reduction in *Pax2* expressing cells and an increase in the population of *Tlx3* expressing cells. However, no change to the Purkinje cell population was seen, in contrast to the *Prdm13^GFP^* mouse study.^12^ Whittaker et al. also evaluated the hypothalamus of adult *Prdm13^Δ^^2^^Δ^^3^* mice and found that *Prdm13* loss results in a reduction of the excitatory *Kiss1* neuron population of the Arc nucleus, but not the anteroventral periventricular nucleus. The loss of these *Kiss1* cells was detectable as early as E14.5.^12^ This finding further demonstrates the role of PRDM13 in the repression of glutamatergic neurons in the brain.

Understanding how PRDM13 regulates development of neurons within the brain is critical as mutations leading to a loss of PRDM13 have been associated with cerebellar hypoplasia and perinatal brainstem dysfunction in humans.^12^^,^^33^ Overall, studies of PRDM13 loss in the development of the vertebrate brain further support that PRDM13 plays a critical role in the cell fate decision between, and patterning of, excitatory and inhibitory neurons (Fig. 5). These studies also determined more specifically that PRDM13 is a key transcriptional regulator for proper development of the *Pax2* and Purkinje neuronal populations of the cerebellum, and the excitatory *Kiss1* population in the Arc nucleus of the hypothalamus.^12^^,^^33^^,^^44^

**Figure 5.**
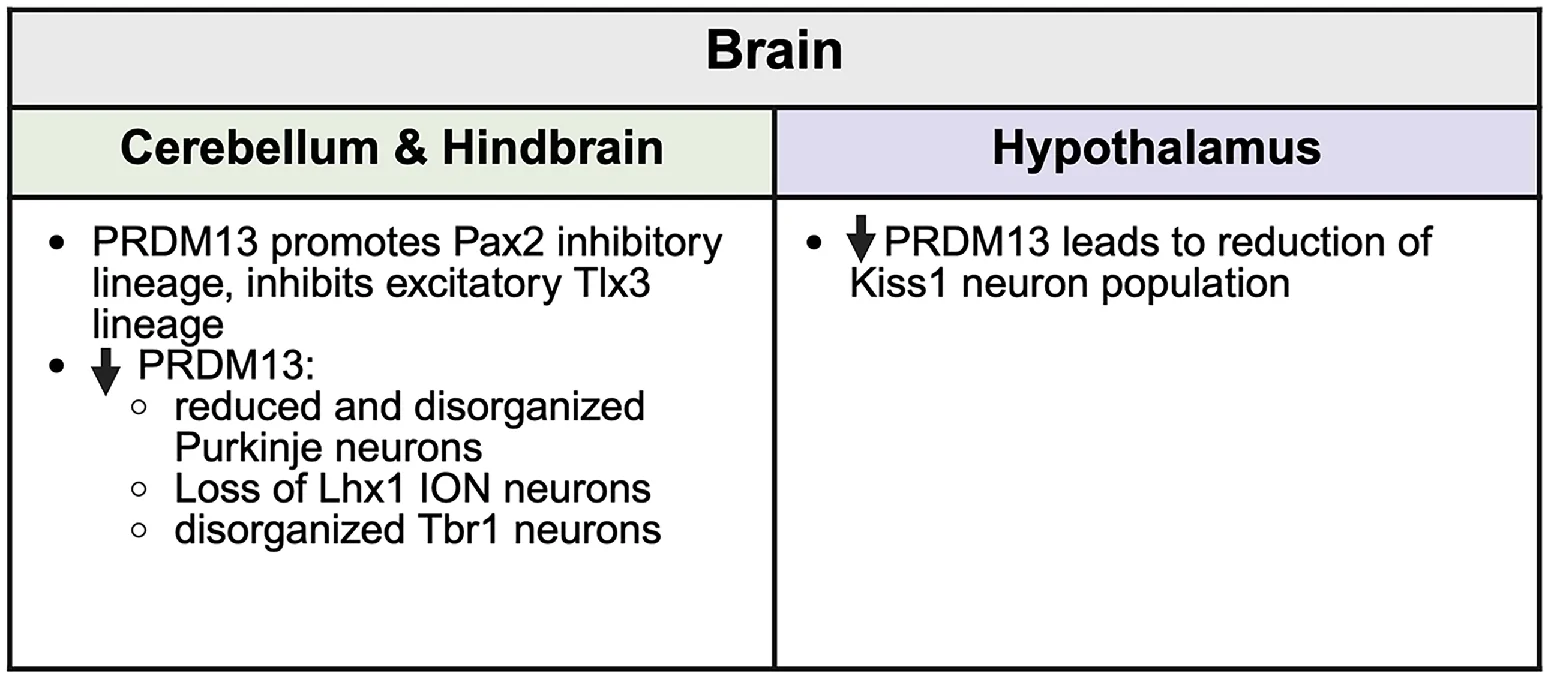
**Summary of PRDM13 function in the developing brain.** PRDM13 is critical for proper neuronal development and patterning in the brain. PRDM13 has been shown to promote specification of inhibitory neurons and the expense of excitatory neurons. Specifically, PRDM13 loss affects specification of Purkinje, Lhx1, and Kiss1 expressing neurons and patterning of Purkinje and Tbr1 expressing neurons.^12^^,^^33^^,^^44^ Figure generated in BioRender (agreement number: GW2A1D6ELX).

## PRDM13 in the Retina

### PRDM13 in Retinal Development

PRDM13 is a particularly interesting gene in the retina as it is expressed throughout retinal development and into adulthood.^11^^,^^31^ *Prdm13* expression within the developing mouse retina begins at approximately E12.5, where it is detected within the neuroblastic layer.^11^ At E13.5, the *Prdm13^fusionGFP^* mice show that all GFP positive cells colocalize with PTF1a.^9^ As PTF1a is known to be present in progenitor cells specifying to either amacrine or horizontal cells within the retina, this PRDM13 and PTF1a double positive population is likely a subset of amacrine progenitor cells.^45^ The colocalization of PRDM13 and PTF1a is consistent with published data from the developing neural tube, where PRDM13 is almost exclusively co-expressed with PTF1a.^8^ Additionally, there is evidence from studies in both the mouse and frog that the PRDM13-PTF1a negative feedback loop first identified in the neural tube is also seen in the developing retina.^9^^,^^27^ By E15.5, the number of PRDM13 and PTF1a double positive cells is reduced to approximately 89%.^9^ At this time, a small number of cells stained with EdU, a marker of the S-phase of the cell cycle, also show detectable PRDM13.^11^ By birth, the number of PRDM13 and PTF1a double positive cells drops to approximately 80%, consistent with the known downregulation of PTF1a in the post-natal retina.^9^^,^^46^

Interestingly, a small percentage of the PRDM13 and PTF1a co-expressing cells also express OTX2, a marker important for bipolar and photoreceptor specification.^47^^–^^49^ Whereas OTX2 positive cells have been shown to specify to amacrine cells, PTF1a positive cells have never been shown to specify to photoreceptors. This indicates that PRDM13 may repress OTX2 in progenitor cells to prevent photoreceptor and bipolar cell specification within these PRDM13, PTF1a, and OTX2 positive cells.^9^ However, the regulation of OTX2 via PRDM13 has not yet been demonstrated and requires further investigation. Even after PTF1a is no longer expressed, PRDM13 expression is maintained in approximately 33% of the amacrine cells of the mature mouse retina, particularly in glycinergic and non-GABAergic non-glycinergic (nGnG) subtypes.^9^^,^^11^ PRDM13 is excluded from cholinergic, dopaminergic, and glutamatergic subtypes.^9^ Co-expression of Prdm13 alongside Ptf1a and EdU has also been demonstrated in retinal progenitor cells within the developing frog retina. Within the mature frog retina, Prdm13 was detected within 40% of glycinergic and GABAergic amacrine cells.^27^ This similarity in expression and localization of PRDM13 across species emphasizes a conserved PRDM13 function for vertebrate development and mature amacrine cell maintenance.

### Loss of PRDM13 in the Retina

As PRDM13 expression during development appears specific to retinal progenitors that will specify to amacrine cells; research on PRDM13 in vertebrate retinal development has focused on amacrine cell fate specification. Within the frog retina, knockdown of Prdm13 using morpholino antisense nucleotides showed a specific loss of glycinergic but not GABAergic amacrine cells.^27^ Knockdown studies conducted with both the *Prdm13^Δ^^2^^Δ^^3^* and *Prdm13^GFP/^^Δ^^115^* mice showed similar overall findings, with as much as a 25% reduction of amacrine cells in the mature retina. The lost amacrine cells in the mature retina are primarily those which express EBF3, CALB1, or CALB2.^9^^,^^11^

To determine if the GABAergic and glycinergic subtypes develop normally then undergo apoptosis, or do not develop at all, investigators looked at developing retinas from the *Prdm13^Δ^^2^^Δ^^3^* and *Prdm13^GFP/^^Δ^^115^* mouse lines. At E17.5 and P2, no change in PAX6 expression, a marker of developing amacrine cells, was observed within the retina. By P5, when more specific subtype markers can be detected, a significant decrease in EBF3 was seen in the developing inner nuclear layer (INL), alongside an increase in the number of PRDM13 cells co-expressing BHLHB5, suggesting that cells which should be specifying the EBF3 amacrine subtype could be taking on other subtype identities.^9^ The normal PAX6 expression but altered subtype specification indicates that amacrine cells as a class are developing properly; however, they do not properly specify into the various cell subtypes.^2^^,^^9^^,^^11^ The authors hypothesize that amacrine cell subtypes which generate an excess of cells, such as the BHLHB5 type, likely undergo apoptosis or culling, leading to the phenotype of a reduced amacrine cell population in the adult retina of PRDM13 knockout mice. In addition to the loss of amacrine cells in the mature retina, investigation using both *Prdm13^GFP^* and *Prdm13^Δ^^2^^Δ^^3^* mice detected disruption of the corresponding amacrine synapse layers of the inner plexiform layer (IPL). In particular, there was overall thinning of the IPL, and loss of the S3 synapsing layer, as shown by staining with CALB2.^9^^,^^11^ Thus, the amacrine subtypes specified by PRDM13 typically synapse within the S3 layer of the IPL.

The *Prdm13^Δ^^2^^Δ^^3^* mouse model was used to assess how the loss of PRDM13 affected spatial, temporal, and contrast sensitivity using optokinetic response (OKR) testing. Contrary to what would be expected with a loss of amacrine cells, *Prdm13^Δ^^2^^Δ^^3^* showed elevated sensitivity compared to wildtype mice.^11^ Based on previous literature which associated OKR to VGLUT3 amacrine cell communication with direction selective ganglion cells, the authors hypothesize that the PRDM13 positive amacrine cells which project to the S2/3 synaptic layer may normally dampen the response of the direction selective ganglion cells, and the loss of these cells then leads to increased OKR.^11^ However, this hypothesis was not further investigated and remains to be studied.

### Overexpression of PRDM13 in the Retina

To complement the work assessing PRDM13 loss of function models, ectopic PRDM13 overexpression was used to examine the role of PRDM13 in retinal health in mice and frogs.^9^^,^^11^^,^^26^^,^^27^ In one study, a plasmid containing *Prdm13* and GFP driven by the ubiquitous CAG promoter was in vivo electroporated into the P0 mouse retina, then harvested at P6 for histological analysis. After 6 days of ectopic *Prdm13* expression, Watanabe et al. saw that 86% of cells expressing GFP also expressed PAX6, whereas only 2% to 5% of GFP expressing cells expressed RHO and CHX10, implying that PRDM13 is sufficient to induce amacrine cell fate specification, particularly those which express CALB1 and CALB2.^11^ Similar results were also noted in the frog retina following prdm13 overexpression. Frog prdm13 and a separate GFP plasmid were co-transfected in vivo into retinal progenitor cells of the developing eye field at stage 18 of development using lipofectamine.^27^^,^^42^ Analysis was performed at stage 41, when retinal cells are actively differentiating, and the authors found that there was an increase in the number of amacrine cells along with a decrease in the number of bipolar cells and Müller glia.^27^^,^^42^

In contrast to these two studies, in vitro induction of ectopic *Prdm13* expression on P0 mouse retinal explants did not show the same increase in amacrine cells. Although Goodson et al. did see that the majority of transfected cells also expressed PAX6, consistent with the other mouse study, they did not see an increase in expression of EBF3 or CALB2, suggesting PRDM13 was not preferentially inducing amacrine cell fate specification.^9^^,^^11^ They did, however, note that many transfected cells did not express PAX6 or OTX2, a sign that the transfected cells may be undergoing apoptosis. They also saw a striking loss in the number of OTX2 positive cells, further indicating that PRDM13 overexpression may be particularly detrimental to developing photoreceptors.^9^ These contrasting results could be due to differences in the method – in vivo versus in vitro ectopic PRDM13 overexpression – particularly as all in vivo experiments, regardless of species, presented with similar findings.

Despite the contrasting data of the PRDM13 overexpression studies, it is clear from the published work that PRDM13 plays a role in amacrine cell development within the retina. In particular, PRDM13 loss of function studies demonstrate that PRDM13 is necessary for proper amacrine subtype specification, but not the development of the amacrine cell class.^9^^,^^11^^,^^27^ There is some evidence to suggest that PRDM13 overexpression could impact other cell types, with the loss of bipolar and Müller glial cells seen in the frog prdm13 overexpression study, and the loss of photoreceptor precursors in both mouse PRDM13 overexpression studies.^9^^,^^11^^,^^27^ These results are particularly interesting as the loss of PRDM13 does not appear to impact the specification of non-amacrine cell types within the retina. How PRDM13 dysregulation alters the finely tuned transcriptional regulatory network for retinal cell fate specification is an important open area for further study (Fig. 6).

**Figure 6.**
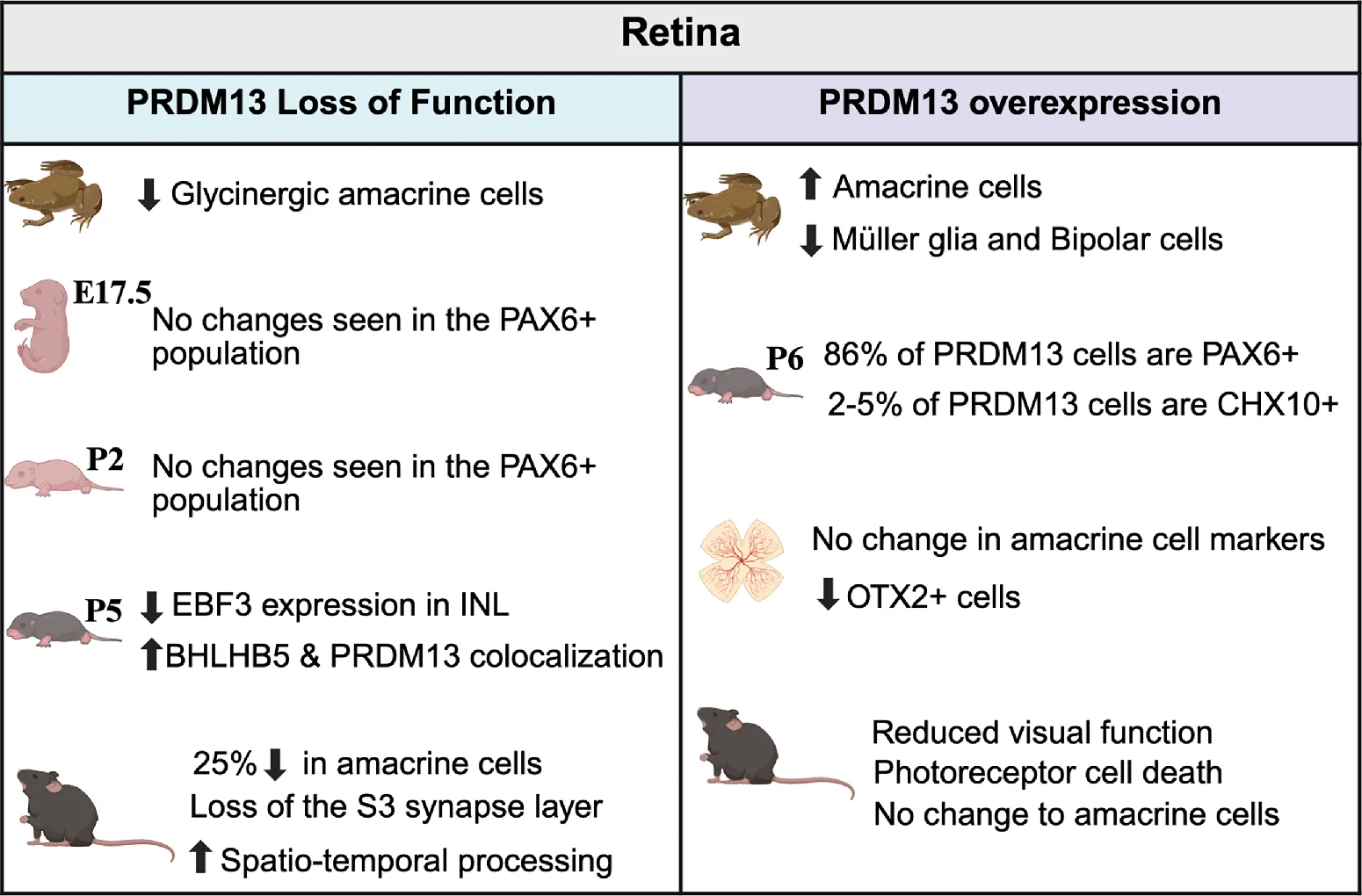
**The impact of PRDM13 dysregulation on the vertebrate retina.** The loss of PRDM13 leads to a reduction in the number of amacrine cells in the mature murine retina due to improper amacrine subtype specification.^9^^,^^11^ This loss of amacrine cells leads to a loss of the S3 synapse and increased optokinetic responses in PRDM13 knockout mice.^9^^,^^11^ The outcome of PRDM13 overexpression studies indicate that PRDM13 overexpression in development causes an increase in amacrine cell specification at the expense of Müller glia, bipolar cells, and/or photoreceptors; whereas PRDM13 overexpression in the mature retina causes photoreceptor specific cell death.^9^^,^^11^^,^^21^^,^^22^ P, post-natal day; E, embryonic day; INL, inner nuclear layer. Figure generated in BioRender (agreement number: UA2A1D6HWB).

### PRDM13 in the Adult Retina

Whereas the majority of the research has focused on understanding the role of PRDM13 during retinal development, *Prdm13* expression is maintained in some mature amacrine cells of the mouse retina.^11^ As previously described, loss of PRDM13 during retinal development leads to improper specification of amacrine cell subtypes within the mature retina.^9^^,^^11^ However, no work has been done to knockout PRDM13 specifically within the mature retina, so it is not yet understood how a loss of PRDM13 during adulthood may affect the retina.

Recent development of the tetracycline-inducible PRDM13-OE mouse model allowed for the study of *PRDM13* overexpression on a conditional basis. With this model, the *Prdm13* locus is unaffected, but *PRDM13* can be overexpressed when the mouse is provided with doxycycline, a tetracycline drug. To date, one study has utilized the PRDM13-OE mouse model to investigate the impact of *PRDM13* overexpression on mature retinal health.^26^ Within this study, it was determined that acute *PRDM13* overexpression for a 24-hour window of time does not lead to reduced retinal function; however, longer durations of overexpression, out to 6 or 10 days, causes significant loss of retinal response to light and photoreceptor-specific cell death. To better understand how *PRDM13* overexpression impacts photoreceptor cell survival, the authors assessed transcriptional changes for key photoreceptor genes during and after overexpression of *PRDM13*. They noted that many genes – such as *Nr2e3*, *Gnb3*, and *Pde6c* – were reduced when *PRDM13* was overexpressed but returned to wildtype levels 3 weeks after *PRDM13* was no longer overexpressed. This work also showed dysregulation of many amacrine cell genes when *PRDM13* was overexpressed; however, this dysregulation did not lead to any detectable changes in the amacrine population as a whole.^26^ As this mouse model ubiquitously overexpresses PRDM13, future investigation with more targeted retina or cell type-specific overexpression will be important to fully understand the mechanism by which PRDM13 dysregulation affects photoreceptor cells.

## Inherited Retinal Dystrophies Associated With PRDM13

Retinal dystrophies which result from genetic mutations fall under the umbrella of inherited retinal dystrophies (IRDs). Due to the rise in affordability and availability of whole genome sequencing technologies, 523 genes have been linked to IRDs.^50^ The distribution of these mutations is approximately 40% missense, approximately 22% frameshift, approximately 18% nonsense, and approximately 15% alternative splicing, with additional in-frame indels and other variants making up the remaining approximately 5%.^51^ These mutations can be found in the genes of autosomal and sex chromosomes, as well as in mitochondrial DNA, and can be inherited in a variety of forms. Over 50 subtypes of IRDs have been described, with the clinical presentation of IRDs ranging from improper retinal development to a slow retinal degenerative process which can span decades.^51^

One type of IRD is maculopathies, which are characterized by either the initial or sole pathology focused on the macular region of the retina. Disruption of the macular region can lead to reduced color and central vision, as well as localized atrophy of the RPE. Although IRDs are typically monogenic diseases, the most common maculopathy is age-related macular degeneration (AMD), a non-monogenetic condition which has been associated with variants in over 100 genes.^52^ Approximately 288 million people are expected to develop AMD by 2040, emphasizing the importance of understanding macular development and health to support therapeutic development.^53^ However, as AMD is a complex disease associated with a large number of genetic and environmental risk factors, it is a challenging disease to study.^52^ This has led some researchers to focus on the investigation of more rare monogenic maculopathies in order to gain an understanding of macular development and maintenance. Three autosomal dominant IRDs –NCMD, PBCRA and CPPCRH – represent a spectrum of macular disease related to the dysregulation of *PRDM13*.^14^^–^^16^^,^^54^^,^^55^ Upon initial examination, these diseases present with the macula being the primary affected area; however, they are clinically distinct, and the genetic mutations governing *PRDM13* expression in each disease are unique, as further described below.

### North Carolina Macular Dystrophy

NCMD was first described in the early 1970s by 3 independent groups studying the same family.^56^ In 1971, Lefler et al. described NCMD as an autosomal dominant macular dystrophy with high variability in phenotype, but normal retinal function on electroretinography (ERG) and color vision tests. In 1974, Frank et al. categorized this high variability into the 3 stages of a progressive disease, and finally, in 1977, Gass et al. coined the term “North Carolina Macular Dystrophy” and reported clinical findings to suggest this was not a progressive condition.^56^ This inconsistency in the definition of NCMD persisted until the early 1990s, where the release of 2 papers by Small et al. provided long-term follow-up studies of members of the original NCMD family.^54^^,^^56^ Over 50 families affected by NCMD have now been identified across the globe.^57^

Clinical examination over the last 5 decades has given a clear picture of the phenotype of NCMD, which is classified into grades (Fig. 7A).^14^^,^^58^^–^^60^ Grade 1 is characterized by the presence of some bilaterally symmetric drusen-like deposits in the central macular region and a visual acuity of 20/20 to 20/30. Grade 2 patients will have confluent, bilaterally symmetric drusen-like deposits in the central macula, often accompanied by RPE atrophy with a visual acuity between 20/25 and 20/60. Grade 3 patients have large macular caldera often with a temporal shelving edge with subretinal fibrosis, and accompanied by choroidal and RPE atrophy, with a visual acuity of 20/40 to 20/200.^14^^,^^58^^–^^60^ One of the most interesting aspects of NCMD is the relatively high visual acuity, particularly in grade 3 patients, given the presence of large macular caldera. This is thought to be due to the development of an eccentric fixation point, also known as a preferred retinal locus (PRL), typically on the nasal side of the macular lesion. It was demonstrated in a cohort of five patients that the fixation stability at this PRL was near that of a healthy patient.^61^ When the PRL is taken into account for patients with NCMD, reduced amplitudes can be seen on multifocal ERG over the area of the macular caldera.^62^

**Figure 7.**
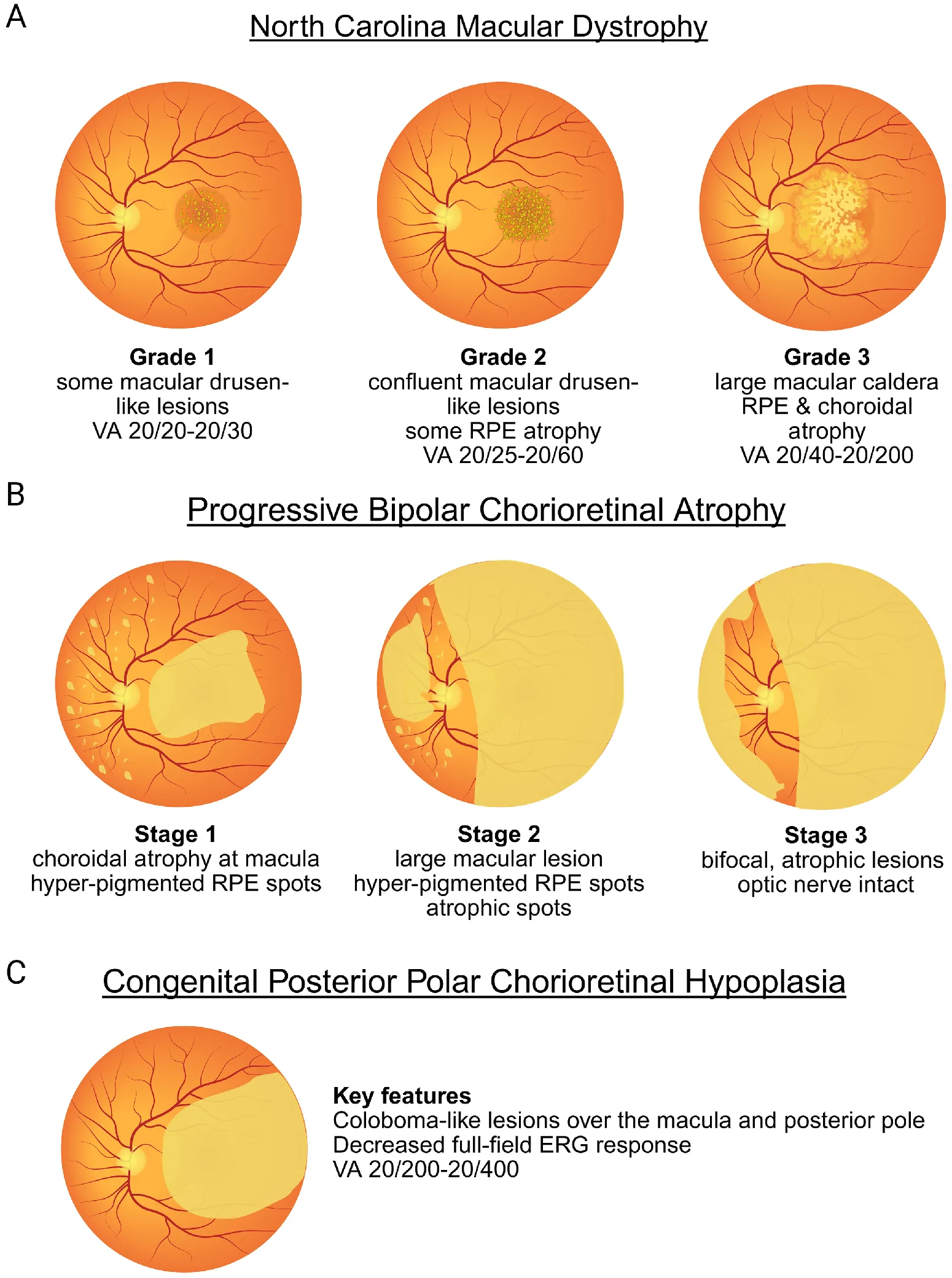
**Presentation of PRDM13-associated macular dystrophies.** (**A**) North Carolina macular dystrophy is a non-progressive condition that presents with three grades of severity. The first two grades are primarily characterized by macular drusen-like deposits, with large macular caldera and atrophy of the RPE and choroid present in grade 3 patients.^58^^–^^60^ (**B**) Progressive bipolar chorioretinal atrophy progresses through three different stages during the course of disease, beginning with atrophy focused at the macula, then extending out to the posterior pole, until eventually only a strip of retina near the optic nerve remains intact.^15^^,^^55^ (**C**) Congenital posterior polar chorioretinal hypoplasia is characterized by coloboma-like lesions over the macula and posterior pole.^14^ Figure generated in BioRender (agreement number: EY2A6NW3MQ).

NCMD is believed to be a developmental condition, due to the number of young children who are diagnosed with the disease. The youngest patient with NCMD was a boy of 34 weeks of gestational age who was diagnosed with grade 2 atrophy of the RPE and choroid.^63^ Thorough investigation has been completed into the possibility of disease progression in these young patients with NCMD, based on the initial classification of NCMD as a progressive maculopathy in 1974.^54^^,^^56^^,^^64^^–^^66^ In one study, approximately 11% of eyes showed some progression of disease. Typically, this is the result of patients with grade 2 lesions developing choroidal neovascularization that occurs between childhood and adolescence.^66^ However, as only a small proportion of patients have been noted to progress during this window, NCMD is now considered to be a generally non-progressive condition.

There are currently 18 genetic mutations which have been identified in patients with NCMD.^15^^,^^16^^,^^34^^–^^37^^,^^67^^–^^74^ Ten of these mutations are large tandem duplications spanning 43 kb to 898 kb. Three of these duplications are found on chromosome 5 and associated with the *IRX1* gene (Table 2); however, it should be noted that smaller duplications in this region of *IRX1* have been found in genomes of patients without NCMD.^16^ Seven tandem duplications are located on chromosome 6 and contain either all or part of the genes *CCNC* and *PRDM13*, as well as multiple non-coding DNASE1 hypersensitivity sites (DHSs). The remaining eight mutations are single nucleotide polymorphisms (SNPs) located in the DHSs between *PRDM13* and *CCNC* on chromosome 6 (Table 3). Interestingly, there is no correlation between genotype and phenotype, as many members of the same family and with the same mutation will exhibit different grades of disease.^54^^,^^56^ However, it has been suggested that patients with mutations on chromosome 5 typically display abnormal color vision, whereas those with mutations on chromosome 6 typically do not.^75^ Although counterintuitive, this separation between genotype and phenotype has been noted in other IRDs, including those caused by mutations in *CERKL* and *NR2E3.*^51^

**Table 2.** Chromosome 5 Mutations

Variant Number	Type of Variant	Nucleotide Change	Chromosomal Position, Hg19	Reference
V5	Tandem duplication	898 kb dup	5: 3587901–4486027	16
V8	Tandem duplication	45 kb dup	5:4391377–4436535	67
V9	Tandem duplication	43 kb dup	5:4396927–4440442	67

Identified mutations of patients with NCMD on chromosome 5.

**Table 3.** Chromosome 6 Mutations

Variant Number	Type of Variant	Nucleotide Change	Chromosomal Position, Hg19	Reference
V1	SNP	G > T	6: 100040906	16,72
V2	SNP	G > C	6: 100040987	16,69,71
V3	SNP	C > T	6: 100041040	16
V4	Tandem duplication	123 kb dup	6: 100020205–100143306	16
V6	Tandem duplication	69 kb dup	6: 99996226–100065137	68
V7	Tandem duplication	98 kb dup	6: 99984309–100082698	35
V10	SNP	A > C	6:100046783	15
V11	SNP	T > C	6:100046804	15
V12	SNP	A > C	6:100040974	70
V13	Tandem duplication	134 kb dup	6:99932464 – 100067110	73
V14	Tandem duplication	56 kb dup	6:100008141 – 100064368	37
V15	SNP	A > G	6:100046940	72
V16	SNP	G > C	6: 100040906	72
V17	SNP	T > C	6:100046790	36
V18	SNP	G > A	6:100046802	36
V19	Tandem duplication	148 kb	6:100019429–100167607	34
V20	Tandem duplication	98 kb dup	6:99983358–100082211	74

Identified mutations of patients with NCMD, PBCRA, and CPPCRH on chromosome 6.

### Progressive Bifocal Chorioretinal Atrophy 

PBCRA was first described in 1968, with further characterization done in 1996 utilizing clinical information from 54 members of the original PBCRA family.^55^ This autosomal dominant retinal dystrophy is characterized by poor visual acuity with bifocal atrophic lesions, which progress gradually over time. Godley et al. noted that the rate of progression was variable between persons of the same age, which led to the development of a three-stage classification system based on disease pathology rather than age (Fig. 7B).^55^ Stage 1 is characterized by the presence of chorioretinal atrophy at the central macula, alongside white deposits and hyper-pigmented RPE at the edge of the macular lesion, the peripheral, and nasal portions of the retina. Stage 2 has larger macular lesions extending beyond the vascular arcades, the white spots begin to appear atrophic, and clumps of RPE hyperpigmentation are noted. Stage 3 is characterized by large bifocal atrophic lesions leaving only a narrow bridge of intact retina near the optic disc.^55^

The genetic cause of PBCRA was identified using whole genome sequencing of PBCRA affected patients from two unrelated families. One shared heterozygous point mutation was identified among both PBCRA affected families: Chr6:100,046,804T > C.^15^ This mutation is located approximately 7.8 kb upstream of the *PRDM13* gene in the same DHS as NCMD mutations V10, V11, V15, V17, and V18.^36^ Both PBCRA and NCMD appear to be congenital retinal dystrophies with evidence of macular lesions and deposits in the basal retina. The youngest patient with PBCRA was diagnosed with stage 1 disease at 4 days old, consistent with NCMD where symptoms present during infancy.^55^ Outside of these pathological similarities, PBCRA is a progressive disease involving the nasal portion of the retina, making it clinically distinct from NCMD. Visual acuity is not correlated to the stage of disease, unlike NCMD which has defined visual acuity ranges incorporated within the definition of each grade.^15^

### Congenital Posterior Polar Chorioretinal Hypoplasia 

CPPCRH was first described in 2019 in a small cohort of 2 patients, with further clinical characterization in a larger cohort of patients and naming of the disease in 2022 (Fig. 7C).^14^^,^^15^ In the 5 adults of the 2022 cohort which underwent ophthalmic examination, all reported nystagmus and poor vision as early as infancy and early childhood. Upon examination, patients exhibited large, coloboma-like lesions involving the entire macula and extending into the posterior pole, with best corrected visual acuity between 20/200 and 20/400. Full-field ERG showed decreased amplitudes in all responses. Two children of this family also underwent ophthalmic examination, and although both presented with the large, coloboma-like lesions at the macula, they did not have nystagmus.^14^^,^^15^

Sequencing of the *PRDM13* gene region in the two patients with CPPCRH from Silva et al. and the five adult family members in Small et al. found a single heterozygous point mutation in the same DHS as the PBCRA mutation and NCMD mutations V10, V11, V15, V17, and V18: Chr6:100,046,783A > C.^14^^,^^15^^,^^36^ In comparison to the other dystrophies associated with PRDM13, CPPCRH is more similar in clinical pathology to PBCRA than to NCMD. The presentation of CPPCRH is more uniform, with all patients displaying large lesions which cause significant vision loss and nystagmus. Whereas the size of the lesion and extent of vision loss in patients with CPPCRH is similar to patients with PBCRA, the lack of bifocal atrophy makes CPPCRH distinct.

### NCMD, PBCRA, CPPCRH Genetic Mutations, and *PRDM13* Regulation

In total, 20 disease-causing mutations have been identified for NCMD, PBCRA, and CPPCRH.^14^^–^^16^^,^^34^^–^^37^^,^^67^^–^^74^ Three of these mutations are located on chromosome 5 and have been associated with the *IRX1* gene (see Table 2), whereas the other 17 are found on chromosome 6 and are associated with the *PRDM13* and *CCNC* genes (Table 3).^74^ As the majority of mutations have been found in chromosome 6, and these mutations span all 3 IRDs, we have focused the next section on the 17 mutations in chromosome 6 (Fig. 8).

**Figure 8.**
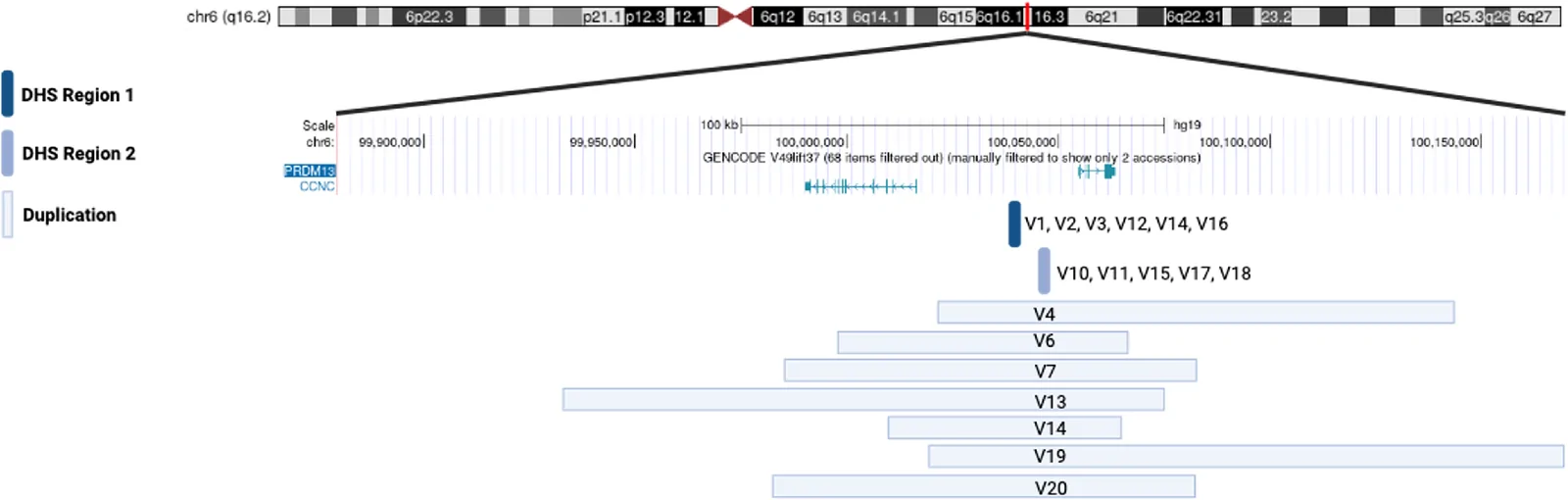
**Map of NCMD, PBCRA,** **and** **CPPCRH mutations on chromosome** **6.** Locations of the 17 NCMD, PBCRA, and CPPCRH mutations located on chromosome 6.^28^^–^^32^^,^^34^^,^^61^^–^^67^ Mapping of *CCNC* and *PRDM13* using the UCSC genome browser (http://genome.ucsc.edu).^77^ Figure generated in BioRender (agreement number: WJ29RI3TJR).

DHSs are regulatory regions of the genome that mark cis-regulatory elements, such as enhancers and promoters, that are vital to proper regulation of gene expression.^76^ The involvement of the pathogenic SNPs falling in non-coding DHSs, and the duplication of these same regions in the tandem duplication mutations – rather than the mutations falling in gene coding regions – posed a new challenge for those looking to determine the mechanism behind NCMD, PBCRA, and CPPCRH. Disruption of these regulatory sites can have a wide range of effects on the genome, from disruption of transcription factor binding sites to changes in topological associated domain (TAD) formation.^36^ Small et al., in their 2016 paper which identified the first 3 SNPs in a DHS and the first tandem duplication for NCMD, focused their investigation onto the nearby genes to these variants, namely *CCNC* and *PRDM13*.^16^ *CCNC* is a known ubiquitous transcriptional regulator associated with cell cycle signaling pathways, whereas *PRDM13*, as previously described, is a transcription factor expressed in neuronal tissues.^16^^,^^18^^,^^19^^,^^77^ Their work utilized human iPSCs differentiated to retinal organoids, where they tested the expression of these two genes throughout retinal development. They found that *CCNC* expression was largely stable between days 0 and 100 of retinal development, but *PRDM13* expression decreased as the organoids matured, implying that *PRDM13* has a critical role in retinal development.^16^ The inclusion of the whole *PRDM13* gene, but not the full *CCNC* gene, in many of the tandem duplications, strengthened the idea that *PRDM13* dysregulation was likely the driver of disease.

Following quickly in 2017, mutation V7 – a large tandem duplication of approximately 98 kb containing the full *PRDM13* and *CCNC* genes as well as the DHS – was identified in a patient with NCMD.^35^ This further strengthened the need to understand whether dysregulation of *PRDM13* or *CCNC* alone is sufficient to drive retinal pathology. To investigate this, Manes et al. utilized the UAS/Gal4 system in flies to individually knockdown or overexpress the fly orthologs of *CCNC* (CycC) and *PRDM13* (CG13296) specifically in photoreceptors.^35^ Knockdown of neither *CCNC* nor *PRDM13* orthologs led to retinal defects in the fly. With overexpression, the *CCNC* ortholog again showed no impact, but the *PRDM13* ortholog led to a striking defect in the eye-antennal imaginal disc and photoreceptor malformation, which the authors likened to a patient with grade 3 NCMD. This work led the authors to conclude that *PRDM13*, rather than *CCNC*, is the driver of the NCMD phenotype.^35^

While it can be intuited that a full duplication of the *PRDM13* gene, such as the tandem duplication variants, would likely result in *PRDM13* overexpression, the association between the SNPs within the DHSs upstream of *PRDM13* and their impact on its regulation and expression are less predictable. Regulatory elements, such as DHSs, are known for their ability to not only regulate nearby genes, but also to have interactions across large regions of the genome.^76^ The identification of the V10 and V11 SNPs, in a second DHS approximately 5.7 kb from the previously identified DHS containing V1, V2, and V3, led to critical insights into the interaction of these upstream DHSs on *PRDM13* regulation.^15^ Epigenomic data, such as assay for transposase-accessible chromatin (ATAC) sequencing, ChIP-seq, and high throughput chromatin conformation capture (Hi-C), can be integrated by in silico analysis using the activity-by-contact (ABC) model to predict enhancer-genome interactions.^78^ Green et al. analyzed adult human retina and macula ATAC-seq and ChIP-seq data sets, as well as Hi-C data from human embryonic stem cells, to identify potential enhancer regions within 100 kb of the *PRDM13* transcriptional start site.^71^ Data from whole retina tissue identified 47 enhancer candidates, whereas macula-specific data identified 53 candidates. V11 fell within an enhancer candidate which was identified in both the whole retina and macula-specific datasets, whereas V1, V2, V3, and V12 fell in an enhancer candidate only present in the macula-specific data.^71^

Two approaches have currently been used to determine what genes these enhancer elements may regulate. The first was the ABC method, previously described, which found both enhancer candidates were predicted to interact with *FLH5*, *SIM1*, and *PRDM13.*^71^ A second method used previously published chromosome conformation capture data from human embryonic stem cells and neural progenitor cells to predict 3-dimensional DNA interactions specifically for the DHS containing mutations V10 and V11. This identified eight genes as possible interaction regions, five of which are genes important for retinal development: *PRDM13*, *FAXC*, *POU3F2*, *TSTD3*, and *USP45.*^15^ Both methods of analysis predict that *PRDM13* is a regulatory target of the mutated DHS.

More direct evidence of *PRDM13* regulation by these DHSs was demonstrated by Van de Sompele et al. in 2022.^72^ The authors utilized a luciferase reporter assay in ARPE-19 cells and in frog embryos to demonstrate that certain suspected *PRDM13* cis-regulatory elements could drive increased expression of their luciferase construct compared control constructs. In particular, they tested a novel proposed enhancer region, as well as the two DHSs which are known to contain the pathogenic SNPs. The authors found that their proposed novel enhancer and the DHS containing V10 and V11, but not the DHS containing V1, V2, V3, and V12, was able to drive luciferase expression in frog embryos at developmental stage 42/43, just as the retinal cells have finished developing.^42^^,^^72^ This expression is consistent with data in ENCODE, the Encyclopedia of DNA elements, which confirmed that the DHS in which mutations V10 and V11 are located, and the authors’ proposed novel enhancer is active, is open and accessible around day 103 of human retinal development; however, the DHS in which V1, V2, V3, and V12 is not open and accessible at this time.^15^^,^^72^

To further understand how *PRDM13* expression may be impacted by the various mutations identified in patients with NCMD, PBCRA, and CPPCRH, Van de Sompele et al. performed an in vitro enhancer assay.^72^ The authors created luciferase reporter constructs each containing one pathogenic SNP or a duplication of the V1, V2, V3, and V12 DHS, or the V10 and V11 DHS. Interestingly, the SNPs in the V1 to V3 DHS showed an increase in luciferase expression of 1.6 to 3.2-fold, whereas the SNPs in the V10 and V11 DHS showed reduced luciferase expression of 0.6 to 0.7-fold compared with their respective controls. Additionally, the 3 constructs mimicking the duplications showed a 0.5-fold luciferase expression compared with respective controls. The results of this assay indicate that the pathogenic SNPs have opposing regulatory effects on *PRDM13* expression, with the V1 to V3 mutations leading to increased *PRDM13* expression, whereas the V10 and V11 mutations, as well as the duplications, reduce *PRDM13* expression. However, as mentioned by the authors, this assay is outside the context of the full genome where these regulatory regions could interact with other regulatory elements or transcription factors not expressed within the in vitro system, which could alter the function of these mutations.^72^

Multiple investigations have been done to predict how the SNPs could alter transcription factor binding sites at the DHSs.^15^^,^^36^^,^^71^^,^^72^ Using UniPROBE, it was found that the V10 mutation led to the gain of a HOXD13 binding site, and the loss of many binding sites for proteins such as EN2 and those in the LHX family. Additionally, the V10 SNP was expected to increase the binding affinity for proteins in the OTX family, including OTX2 and CRX, proteins known to be important for photoreceptor development.^15^ Use of the TRANSFAC database also suggests that the V10 mutation affects OTX family binding sites.^72^ TRANSFAC also predicted the loss of POU family binding sites with the V10 mutation, specifically for POU2F1 and POU2F2, which are known to be important regulators of cone photoreceptor development.^72^ The V11 mutation is known to generate new binding sites for multiple proteins including BHLHB2, TCEFC, SMAD3, and CBF1.^15^ TRANSFAC has further been used to predict changes from the V3 and V12 mutations, which are expected to create OTX family and PAX6 binding sites, respectively.^72^

The interpretation of these investigations into the mutations identified on chromosome 6 suggest that spatiotemporal dysregulation of the *PRDM13* gene is the cause of NCMD, PBCRA, and CPPCRH. The range of mutations, when the chromatin is open and accessible, and the unique impact the mutations have to alter transcriptional binding sites or changes in TAD organization, could explain the broad range of phenotypes present within this spectrum of inherited maculopathies. However, further investigation is needed to directly link PRDM13 dysregulation to these human IRDs.

## Conclusions and Future Investigations

Development of the vertebrate retina requires a complex regulatory network which is executed by a variety of transcription factors capable of promoting or inhibiting genes important for specifying cell fate and organization.^5^^–^^7^ Although the regulatory functions of some transcription factors are well understood, others, including the PRDM family, remain unclear.^45^^,^^79^^–^^83^ In the case of PRDM13, it is well established that this transcription factor plays a critical role in neuronal development throughout the central nervous system (Fig. 9).^8^^,^^9^^,^^12^ PRDM13 is important for ensuring the proper balance between inhibitory and excitatory neuron specification as well as proper localization of these cells within a tissue.^10^^,^^12^^,^^44^ These functions seem to be conserved across the neural tube, cerebellum, and retina. The direct regulatory targets of PRDM13 in the dorsal neural tube were identified using ChIP-seq and include many bHLH genes, as well as genes involved in cell cycle and proliferation.^10^ PRDM13 is believed to perform its regulatory functions by acting as a transcriptional repressor either on its own or through recruitment by other transcription factors, but it still remains to be studied whether PRDM13 could additionally act as an activator during development.

**Figure 9.**
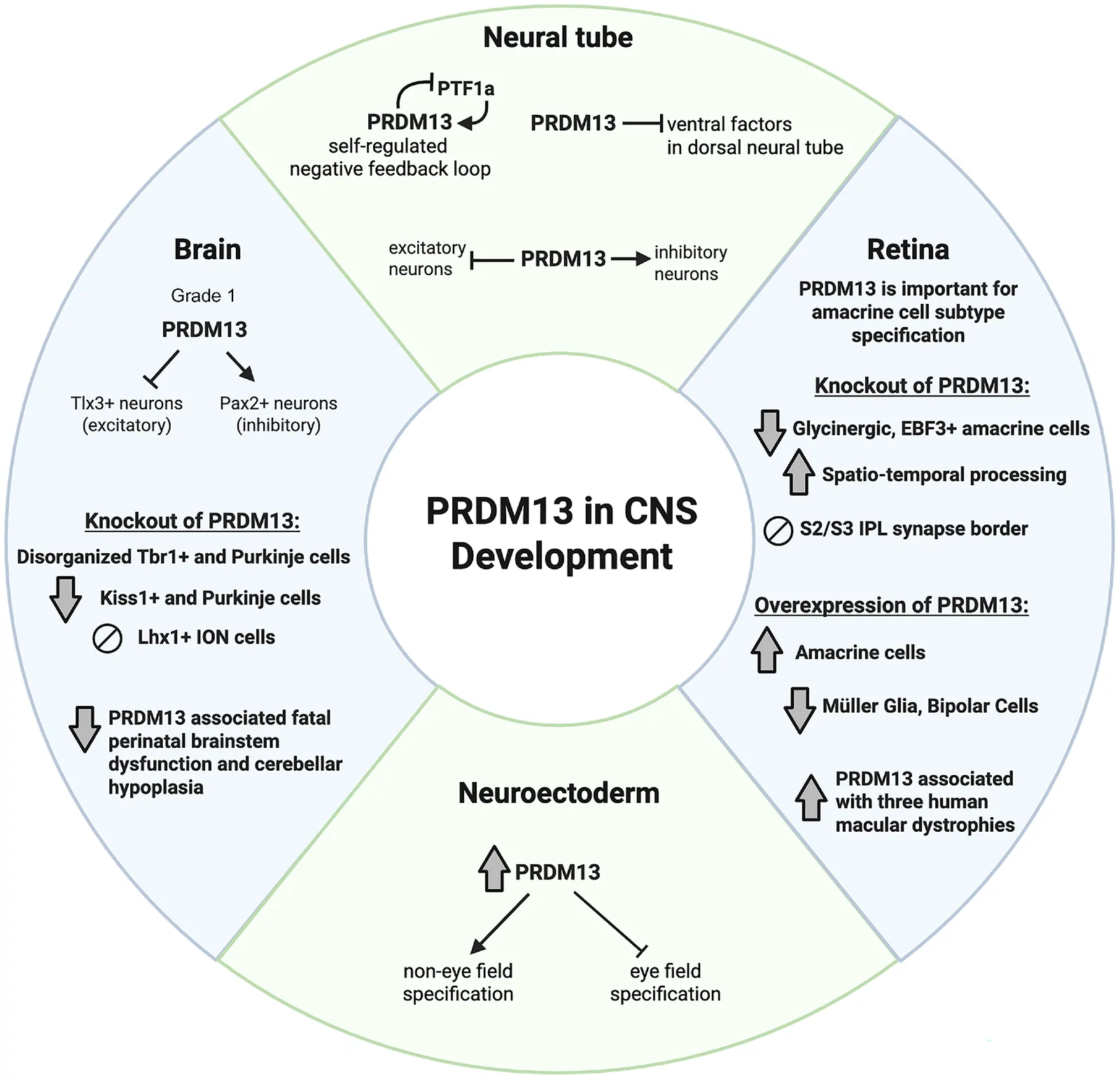
**Graphical summary of PRDM13’s role in the central nervous system.** Summary of the body of research investigating the role of PRDM13 in the central nervous system from development through adulthood in multiple model systems. Figure generated in BioRender (agreement number: JD2A1DLE90).

A key discrepancy that remains in understanding the function of PRDM13 is whether or not the PR domain conveys HMT activity. Whereas the majority of research conducted has suggested that the PR domain is not necessary for PRDM13 function, the assays have primarily assessed outcomes related to neuronal specification and expression levels of downstream gene targets following expression of truncated PRDM13 constructs in mouse and chick neural tube.^8^^,^^10^ However, in an assay specifically designed to test enzymatic HMT activity following ectopic expression of the full length PRDM13 in HEK293 cells, 3H-SAM incorporation on core histones, specifically H3K9 and H3K27 trimethylation, was found to be increased over their vector control.^31^ These conflicting results could indicate a species-specific difference in PRDM13 function because the only positive HMT data have come from human cells, a tissue-specific difference as HEK293 cells are not a neuronal cell type, or that PRDM13 HMT activity may not be important for neuronal cell specification but for another currently unknown function. The field would benefit from replication of the HMT activity assay performed in HEK293 cells, as well as performed within neuronal cells of multiple species. Ideally, both full length and truncated protein constructs would be tested to determine the specificity of any PRDM13-related HMT activity.

As PRDM13 is expressed in multiple neuronal tissues, it is of interest to understand tissue-specific functions of PRDM13. There is some evidence to indicate that PRDM13’s functional domains may vary between tissues.^10^^,^^32^ Basinski et al. demonstrated that the first zinc finger domain of PRDM13 is necessary for the repression of eye-field mNEO differentiation.^32^ However, investigation into dorsal neural tube development in *Prdm13^Δ^^ZF^* mice, which have only the PR/SET domain and the first zinc finger domain, were reported to phenocopy the *Prdm13^GFP^* knockout mouse strain.^10^ Together, these data imply that the first zinc finger domain conveys sufficient function to PRDM13 to allow it to negatively regulate eye-field mNEO differentiation but does not convey function to PRDM13 in the dorsal neural tube. Tissue-specific differences in which domains convey function to PRDM13 opens the question of whether PRDM13 regulatory networks are conserved across tissues or if there are tissue-specific activity which have yet to be determined.

One critical avenue to understand tissue specific differences will be the use of ChIP-seq, CUT&RUN, or similar experimental methods to identify the PRDM13 binding motif and potential regulatory targets across tissues. In their review of the PRDM family, Di Tullio et al. used the Princeton “DNA-binding Specificities of Cys_2_His_2_ Zinc Finger Proteins” DNA binding site predictor to predict the PRDM13 binding motif.^17^ The site predicted a binding motif of CxAGTCCGC; however, this binding motif has not been experimentally validated.^17^ Although it is possible that the PRDM13 binding motif may be conserved across tissues, based on discrepancies in the necessity of the first zinc finger domain between the developing dorsal neural tube and eye field discussed above, there is a high probability of tissues having differing motifs. Particularly for the retina, it would also be of interest to apply this idea across developmental time, to understand how the binding motif, and therefore regulatory targets, of PRDM13 change as the retina matures and its expression is restricted to the amacrine cell lineage.

Whereas the data from PRDM13 overexpression studies are more varied, there is new promising evidence from recent literature to suggest that PRDM13 overexpression may preferentially induce amacrine cell specification at the expense of bipolar cells, Müller glia, and photoreceptor precursors, and that PRDM13 overexpression in the mature retina leads to photoreceptor-specific cell death.^11^^,^^26^^,^^27^ These exciting data emphasize the need for further investigation into the regulatory network downstream of PRDM13, to provide critical insights into its role in retinal development and retinal health.

As current research into the natural expression pattern of PRDM13 predominantly highlights its role in amacrine cell fate specification, it still remains unclear how or why PRDM13 dysregulation preferentially affects macular development.^9^^,^^11^^,^^27^ The most compelling evidence demonstrating PRDM13’s utility in macular development comes from in silico work investigating regulatory elements governing PRDM13 expression.^15^^,^^36^^,^^71^^,^^72^ Analysis of publicly available data from human embryonic retina determined that potential PRDM13 regulatory regions, including the regulatory element which contains mutations V10 and V11, are open at day 103 of human embryonic development – a key timepoint for macular development.^15^^,^^72^ Additionally, the ABC in silico model predicts an enhancer region spanning the location of four NCMD associated mutations, and this enhancer was predicted to only be present in macular tissue. Another enhancer element which contained a mutation linked to PBCRA was predicted to be in both the macula and full retina.^71^

As the mutations within the predicted PRDM13 regulatory elements are expected to alter transcription factor binding sites, it is possible that one or more of these disrupted binding sites is important for regulating PRDM13 expression within specific cell types.^15^^,^^36^^,^^72^ For example, genes in the POU family were found to be associated with PRDM13 binding sites in ChIP-seq of the mouse neural tube.^10^ The disruption of binding sites for POU family members, while strengthening binding affinity for OTX2, are known to control the timing of photoreceptor development in the mammalian retina.^47^^,^^49^^,^^84^ Within the developing human retina at day 82 and day 125, two transition populations are present, one between neurogenic cells and mature amacrine cells marked by PRDM13, and the other between neurogenic cells and mature photoreceptor cells marked by FABP7.^40^ As these 2 transition populations are both present at the time when the regulatory element containing disease-associated mutations is open at day 103, it is possible that the altered binding sites of these typically photoreceptor-promoting factors could lead to ectopic PRDM13 expression within the neurogenic to photoreceptor transition population, potentially pushing these cells toward an amacrine cell fate or leading to cell death.

The developmental timing of endogenous PRDM13 expression within the retina and the fact that the disease-associated mutations fall within non-coding regions indicate that the NCMD, PBCRA, and CPPCRH mutations prevent proper transcription factor binding at regulatory elements. These elements are open during a key time of human macular development, likely leading to improper PRDM13 expression, and ultimately affecting cell fate specification from which the retina is unable to recover. This hypothesis aligns well with the non-progressive nature of NCMD and CPPCRH, as this dysregulation would only occur for the window of time in which the regulatory region is open. As each mutation was found to affect the binding sites of transcription factors differently, and the extent to which each mutation altered PRDM13 expression was found to be variable, this hypothesis also explains the variable phenotypes seen across the PRDM13 associated maculopathies.^15^^,^^36^^,^^71^^,^^72^

One drawback to this hypothesis is that if these enhancer regions are only open for a short window of time during retinal development, it would not explain the progressive nature of PBCRA in the mature retina. Prolonged PRDM13 overexpression during adulthood has been shown to cause photoreceptor-specific cell death in mice.^26^ This finding highlights the possibility that PBCRA-associated mutations may alter the time in which the regulatory element is open rather than, or in addition to, altering transcription factor binding sites, causing PRDM13 dysregulation into adulthood. Further investigation into the temporal accessibility of the predicted regulatory regions using gene-edited or patient-derived human retinal organoids containing the NCMD, PBCRA, and CPPCRH mutations would provide critical insights into PRDM13 temporal dysregulation and the progressive nature of PBCRA within the mature retina. Additionally, as the macula is a retinal feature not present in most model systems, experimental validation of the influence of these regulatory elements on macular development is challenging.^85^ Although they do not form a true macular structure, research investigating chromatin conformation and regulatory regions in cone-dominant human retinal organoids may be a promising tool to advance our understanding of cone photoreceptor regulatory networks.^85^

Currently, NCMD, PBCRA, and CPPCRH are considered a spectrum of PRDM13-associated diseases; however, there remains debate as to whether these are truly individual diseases or a variable presentation of one disease. From a clinical perspective, the difference in presentation, particularly the progressive nature of PBCRA, favors the classification of these conditions as clinically distinct.^14^^,^^15^ However, as mutations from multiple conditions can reside in the same DHS and are believed to cause PRDM13 dysregulation resulting in disease, a genetic or mechanistic basis could be used to justify classifying these conditions as one disease of variable severity.^15^

One complication to the use of a genetics-based classification approach is the fact that some patients with NCMD have mutations on chromosome 5 which has been associated with dysregulation of *IRX1* rather than *PRDM13*.^67^^,^^86^^,^^87^ Based on clinical presentation, these patients have been diagnosed with NCMD; however, no direct link between IRX1 or the mutated regions on chromosome 5 with PRDM13 has been demonstrated.^67^^,^^72^^,^^86^^,^^87^ Use of a genetics-based classification approach for PRDM13-associated diseases would require the separation of those patients with mutations on chromosome 5 from NCMD to a new, IRX1-associated maculopathy in order to redefine NCMD, PBCRA, and CPPCRH as one PRDM13-associated maculopathy. Further studies focused on understanding the direct regulatory targets and interactors of both PRDM13 and IRX1, as well as how the various genetic mutations, including those on chromosome five, may affect the regulation of PRDM13 will provide valuable insights into the appropriate classification for NCMD, PBCRA, and CPPCRH as one or multiple diseases. Overall, future studies into the regulation of PRDM13 and its downstream regulatory network will be important to provide critical insights into cell fate decisions in the developing retina and how the disruption of these decisions leads to disease.
